# Genetic Causes and Ankle Instability in Hypermobile Ehlers–Danlos Syndrome (hEDS): An Integrated Analysis Using Whole-Exome Sequencing and Stress Imaging

**DOI:** 10.3390/jcm15103881

**Published:** 2026-05-18

**Authors:** Jae-Yoon Kim, Ho-Yeon Lee, Seon-Yeop Kim, Sun-Woo Lee, Minjoo Cho, Jeong Woen Shin, Yeo-Gyeong Yoon, Jinhyuk Lee, Yea Eun Kang, Da Hyun Kang, Seong Kyeong Jo, Chan Kang, Namshin Kim, Jae Hwang Song

**Affiliations:** 1 Genomic Medicine Research Center, Korea Research Institute of Bioscience and Biotechnology (KRIBB), Daejeon 34141, Republic of Korea; jaeyoonkim@kribb.re.kr (J.-Y.K.); tjsdnlswbi5@kribb.re.kr (S.-Y.K.); tjsduq1173@kribb.re.kr (S.-W.L.); whalswn215@kribb.re.kr (M.C.); jwshin0727@kribb.re.kr (J.W.S.); durud103@kribb.re.kr (Y.-G.Y.); 2 Department of Bioinformatics, Korea Research Institute of Bioscience and Biotechnology (KRIBB), School of Bioscience, University of Science and Technology (UST), Daejeon 34141, Republic of Korea; hoyeonlee0831@gmail.com (H.-Y.L.); jinhyuk@kribb.re.kr (J.L.); 3 Department of Laboratory Medicine, Seoul National University Hospital, Seoul National University College of Medicine, Seoul 03080, Republic of Korea; 4 Genome Editing Research Center, Korea Research Institute of Bioscience and Biotechnology (KRIBB), Daejeon 34141, Republic of Korea; 5 Research Center for Endocrine and Metabolic Disease, Integrated Disease Research Institute, College of Medicine, Chungnam National University, Daejeon 35015, Republic of Korea; yeeuni2200@gmail.com (Y.E.K.); ibelieveu113@cnuh.co.kr (D.H.K.); 6 Department of Internal Medicine, Chungnam National University, Daejeon 35015, Republic of Korea; 7 Department of Orthopaedic Surgery, Konyang University Hospital, Daejeon 35365, Republic of Korea; joseongkyeong@gmail.com; 8 Department of Orthopaedic Surgery, Chungnam National University, Daejeon 35015, Republic of Korea; faschan@hanmail.net

**Keywords:** ankle instability, exome sequencing, genetic predisposition to disease, hypermobile Ehlers–Danlos syndrome, stress ultrasonography

## Abstract

**Background**: Hypermobile Ehlers–Danlos syndrome (hEDS), the most common EDS subtype, is characterized by chronic pain and joint laxity, yet no definitive causative genes or imaging-based diagnostic criteria have been established. This study investigated the genetic basis of hEDS using whole-exome sequencing (WES) and objectively evaluated ankle instability. **Methods**: We conducted an observational cohort study with a case–control comparison, including 22 patients and a three-generation Korean family (six individuals, four affected) diagnosed with hEDS by the 2017 criteria. WES was performed; ankle laxity was assessed by the anterior drawer test (ADT), stress ultrasonography, and stress radiography. Healthy young adults (*n* = 24, Beighton score < 5) from our previous study served as controls. **Results**: The hEDS cohort had a mean Beighton score of 8.5, with all participants reporting a family history of hypermobility and musculoskeletal complications. Family-based WES identified variants in *CD44* (c.1516 + 1G > A), *ITIH2* (c.783C > G), and *ADAM21* (c.397C > T) in all affected individuals. In 22 unrelated patients, 114 variants in 103 candidate genes were identified; 17 patients harbored variants in genes from the same pathways as the family-derived causative genes. Compared with controls, the hEDS group showed significantly greater manual ADT grade, anterior talofibular ligament (ATFL) length at rest and under stress, dynamic ATFL change, anterior talar translation, and talar tilt. **Conclusions**: These findings provide molecular evidence that hEDS is a multifactorial disorder involving interconnected biological pathways, and confirm ankle instability as a clinically meaningful diagnostic feature. These complementary approaches may improve diagnostic accuracy and provide insights into the prognosis and therapeutic strategies for hEDS.

## 1. Introduction

The Ehlers–Danlos syndromes (EDS) are a clinically and genetically heterogeneous group of heritable connective tissue disorders (HCTDs) characterized by joint hypermobility, skin hyperextensibility, and tissue fragility [1]. The 1988 Berlin Nosology recognized 11 subtypes, defined by Roman numerals, based on clinical findings and mode of inheritance [2]. However, subjective interpretation of several semiquantitative clinical signs, such as joint hypermobility, skin hyperextensibility, tissue fragility, and bruising, led to clinical uncertainty, diagnostic confusion regarding EDS subtype, and inclusion of phenotypically similar conditions under the broad diagnosis of EDS [1]. Since then, additional EDS subtypes have been described, and with the advent of next-generation sequencing (NGS), pathogenic variants have been identified in collagen-encoding genes and genes encoding collagen-modifying enzymes [1]. Accordingly, the International EDS Consortium proposed a revised classification in 2017, which recognizes 13 subtypes of EDS (Table 1) [1].

Among the 13 subtypes, the diagnosis of hypermobile EDS (hEDS) remains clinical because no reliable or widely applicable genetic etiology has been identified in the vast majority of patients [1]. This likely reflects marked genetic heterogeneity and variable syndromic presentation according to age and sex [1]. Because there is currently no gold-standard laboratory test to confirm or exclude the diagnosis, hEDS should be diagnosed on the basis of clinical criteria alone [1]. Among these criteria, the Beighton score is the most widely used and recognized tool for assessing generalized joint hypermobility (GJH), a key feature of hEDS, by evaluating joint mobility at multiple sites on a 9-point scale [1,3]. However, the Beighton score has several limitations. Range of motion and the distribution of joint hypermobility are strongly influenced by age, sex, and ethnicity [4]. In addition, GJH is not joint-specific, and not all patients with GJH exhibit laxity in the same joints [5]. As a result, diagnosing hEDS solely on the basis of clinical criteria may lead to misdiagnosis, which can substantially affect treatment decisions and the prognosis of joint disease [6,7]. Therefore, there is a clear need for more reliable assessments of hEDS. Identification of the genetic causes of hEDS may also contribute to the development of more definitive, mechanism-based treatment strategies.

Orthopedic surgeons frequently encounter patients with hEDS because of chronic pain and joint laxity [8,9,10]. Patients with joint laxity may present without symptoms, but many develop a range of musculoskeletal problems such as joint pain, recurrent dislocations, tendon disorders, and ligament injuries [11]. Even when no symptoms are evident, increased joint laxity is associated with a substantially higher likelihood of future musculoskeletal injury [12]. For example, hypermobility of the first ray is one of the factors that induces hallux valgus and can be caused by technical mistakes in ballet practice [13].

In particular, patients with hEDS often present with ankle disorders, including chronic lateral ankle instability (CLAI). Previous studies have shown that hEDS is associated with poor clinical outcomes and recurrent instability after ligament repair for CLAI [6,7]. Despite this clinical relevance, current diagnostic assessment of hEDS does not adequately reflect ankle-specific laxity, as the Beighton score is weighted toward the upper limbs and does not directly evaluate the ankle joint [14]. Therefore, additional objective measures are needed to better characterize ankle involvement in patients with hEDS.

We hypothesized that hEDS has identifiable genetic causes that can be detected through whole-exome sequencing (WES), and that patients with hEDS exhibit characteristic ankle abnormalities that can be objectively assessed using stress ultrasonography and stress radiography. Although no reliable genetic etiology has yet been established for most patients with hEDS [1], WES-based analysis combining a three-generation hEDS pedigree with an independent cohort of 22 hEDS patients may help identify candidate genes related to connective tissue integrity. In addition, although stress ultrasonography and stress radiography are widely used to evaluate mechanical ankle instability, no systematic study has comprehensively evaluated these imaging findings in patients with hEDS. From this perspective, the present study aimed to investigate the genetic causes of hEDS through integrated WES analysis and to evaluate ankle status in patients with hEDS using stress ultrasonography and stress radiography, thereby linking molecular findings with objective biomechanical phenotyping.

## 2. Materials and Methods

### 2.1. Ethics Approval, Consent to Participate, and Participant Recruitment

**Figure 1:**
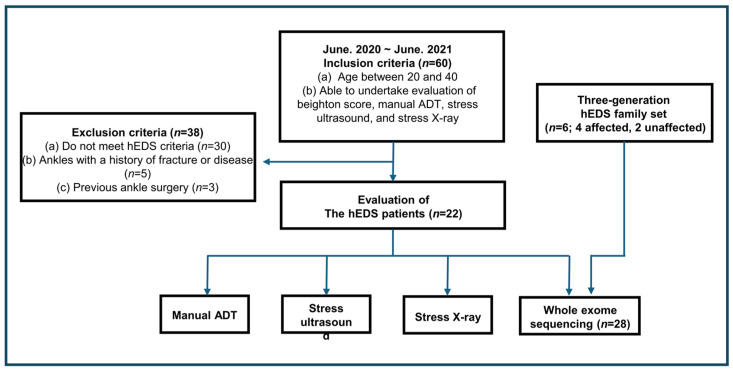
Flow diagram of participant selection and eligibility. A total of 60 individuals were initially recruited through a public announcement. Participants who did not meet the clinical diagnostic criteria for hypermobile Ehlers–Danlos syndrome (hEDS), had a history of ankle fracture or disease, or had undergone previous ankle surgery were excluded. Ultimately, 22 participants were included in the hEDS group. In addition, a three-generation Korean family with hEDS (*n* = 6) was included to investigate the genetic causes.

This observational cohort with control comparison study was approved by the Institutional Review Board of Konyang University Hospital (protocol number: 2019-09-006-003), and written informed consent was obtained from all participants. Participant recruitment was conducted through a public announcement at our hospital, targeting individuals visiting the foot and ankle clinic between June 2020 and June 2021 (Figure 1). Participants were enrolled consecutively and underwent evaluation irrespective of ankle or foot symptoms, including CLAI. The inclusion criteria were as follows: (a) age between 20 and 40 years and (b) ability to undergo evaluation including the Beighton score, manual anterior drawer test (ADT), stress ultrasonography, and stress radiography. Following initial recruitment, participants were screened using the following exclusion criteria: (a) failure to meet the clinical diagnostic criteria for hEDS, (b) history of ankle fracture or disease, and (c) prior ankle surgery. Ankle instability was assessed using manual ADT [15], stress ultrasonography [3], and stress radiography [16]. All clinical examinations and ultrasound assessments for the diagnosis of hEDS were performed by a board-certified orthopedic surgeon (JHS) with 12 years of clinical experience, including 11 years in musculoskeletal ultrasound imaging [3,17]. For comparative analysis, data from a control group (non-GJH group, *n* = 24) obtained from a previous study [3] were used. The control group comprised 18 females and 6 males, with a mean age of 26.9 years (range, 20–40 years). The mean Beighton score in the control group was 1.42.

To investigate the molecular and genetic basis of hEDS, next-generation sequencing analysis using WES was performed. In addition to the study participants, a three-generation Korean family with hEDS (total *n* = 6; affected *n* = 4; unaffected *n* = 2) was included to facilitate identification of potential causative variants (Figure 1; Supplementary File S3: Supplementary Table S1). Family members were diagnosed with hEDS using the same criteria applied to the other participants. Blood samples for WES were collected under approval of the same Institutional Review Board, and all participants provided written informed consent. All procedures were conducted in accordance with the Declaration of Helsinki.

### 2.2. Diagnosis of hEDS

The clinical diagnosis of hEDS requires the simultaneous presence of criteria 1, 2, and 3 [1].

**Figure 2:**
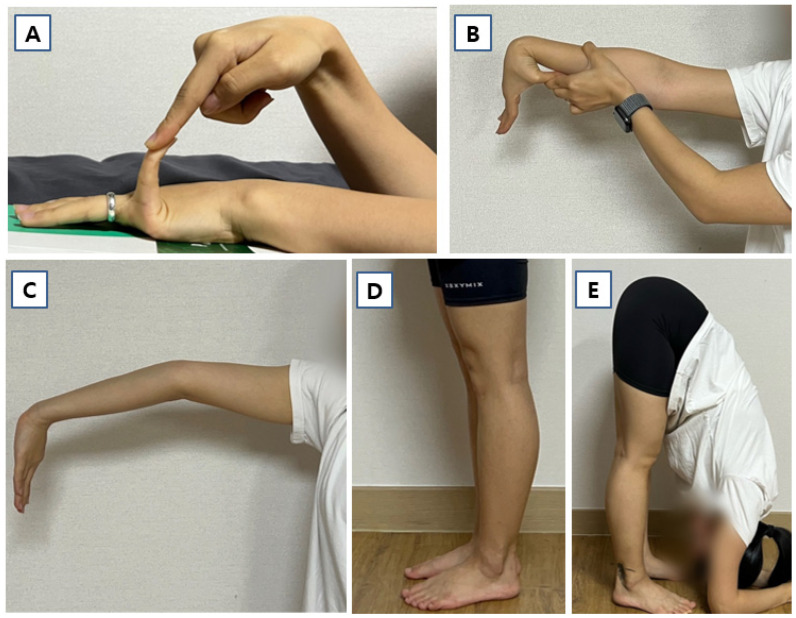
The Beighton score measured in a 20-year-old dancer diagnosed with hEDS. Each joint was measured using a goniometer, and each side was scored independently as outlined. (**A**) With the palm of the hand and forearm resting on a flat surface and the elbow flexed at 90°, hyperextension of the fifth metacarpophalangeal joint beyond 90° relative to the dorsum of the hand was considered positive (1 point). (**B**) With the arms outstretched forward and the hand pronated, passive apposition of the thumb to the ipsilateral forearm was considered positive (1 point). (**C**) With the arms outstretched to the side and the hand supinated, elbow hyperextension beyond 10° was considered positive (1 point). (**D**) While standing with the knees locked, knee hyperextension beyond 10° was considered positive (1 point). (**E**) With the knees fully extended and feet together, the ability to place both palms flat on the floor just in front of the feet was considered positive (1 point). The total possible score was 9.

Criterion 1. Presence of GJH [1]. GJH was defined as a Beighton score of ≥5 out of 9 (Figure 2).

Criterion 2. At least two of the following features must be present (see Supplementary File S1: Supplementary Note S1 for details): (a) systemic manifestations of a generalized connective tissue disorder; (b) positive family history in one or more first-degree relatives; and (c) musculoskeletal complications.

Criterion 3. All of the following prerequisites must be met: (a) absence of unusual skin fragility suggestive of other types of EDS; (b) exclusion of other heritable and acquired connective tissue disorders, including autoimmune rheumatologic conditions; and (c) exclusion of alternative diagnoses associated with joint hypermobility due to hypotonia and/or connective tissue laxity.

### 2.3. Whole Exome Sequencing with Library Preparation

WES was performed using genomic DNA extracted from 2 mL of whole blood. Exome libraries were prepared using the Agilent SureSelect Target Enrichment System (Agilent Technologies, Santa Clara, CA, USA) for Illumina paired-end sequencing (Version C2, December 2018) with 1 μg of input DNA, and the SureSelect Human All Exon V6 probe set was used for all samples. DNA quantity and quality were assessed using PicoGreen (Thermo Fisher Scientific, Waltham, MA, USA) and agarose gel electrophoresis. Genomic DNA was fragmented to a target size of 150–200 bp using a Covaris LE220 ultrasonicator (Covaris, Woburn, MA, USA) according to the manufacturer’s instructions, followed by end repair, A-tailing, adapter ligation, and PCR amplification. For exome capture, 250 ng of the DNA library was hybridized with capture probes at 65 °C for 24 h, and the captured DNA was subsequently washed and amplified. The final libraries were quantified using the KAPA Library Quantification Kit (Roche, Basel, Switzerland) and assessed using the Agilent TapeStation D1000 system (Agilent Technologies, Santa Clara, CA, USA). Sequencing was performed on the Illumina HiSeq 2500 platform (Illumina, San Diego, CA, USA).

### 2.4. Germline Variant Identification

**Figure 3:**
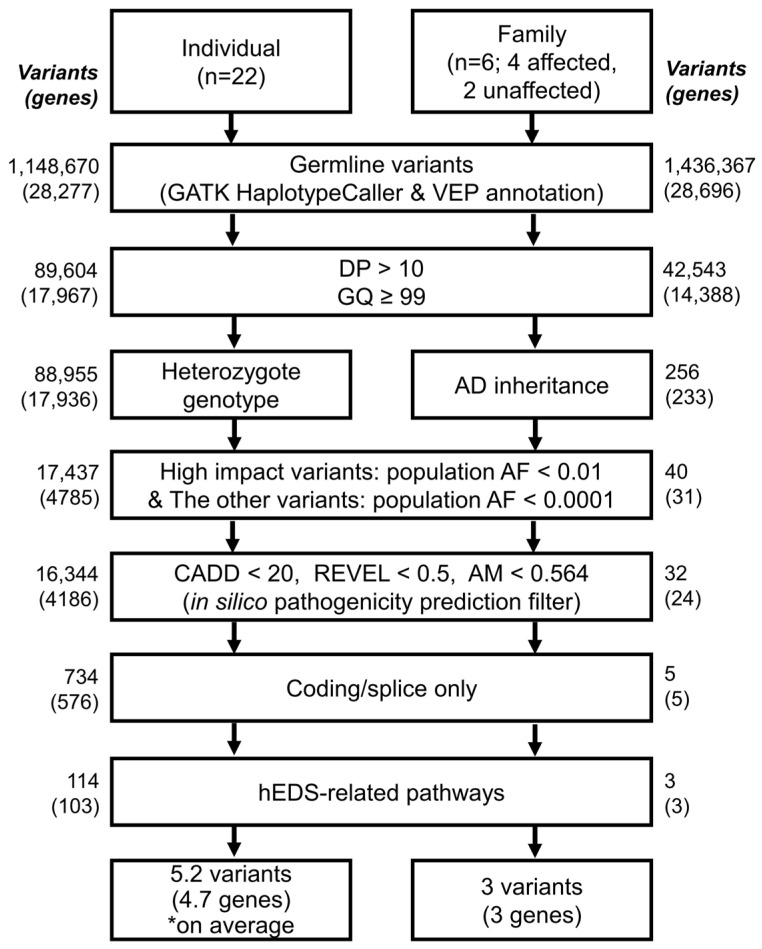
Workflow of integrated NGS analysis in 22 participants and a three-generation Korean family with hEDS. At each filtering step, the number of retained variants is shown, with the corresponding number of genes indicated in parentheses below. In the 22-participant dataset, the reported numbers represent the union of all variants and genes identified across the patients, and the final panel shows the average numbers of variants and genes detected per participant (denoted by an asterisk, *). In the family dataset, the reported numbers represent the union of all variants and genes identified across the four affected individuals, and the final panel shows the variants and genes shared throughout the pedigree.

WES reads were aligned to the GRCh38.p14 reference genome using bwa-mem2 (v2.2.1) [18]. The aligned reads were processed following GATK (v4.3.0.0) best practices [19], including duplicate removal, base recalibration, and gVCF generation using the HaplotypeCaller option. Individual gVCF files were merged into a single cohort-level VCF using the GenomicsDBImport and GenotypeGVCFs options. Variant filtering was performed using the VariantFiltration and SelectVariants options of GATK with the following thresholds: variant quality score (QUAL) < 10.0, quality by depth (QD) < 2.0, mapping quality (MQ) < 20.0, and Fisher strand bias (FS) > 200.0. The filtered variants were subsequently annotated using Ensembl VEP (v115) [20]. Annotation included gene region, allele frequency in population databases, pathogenicity prediction scores, and clinical significance, based on gnomAD v4.1 allele frequency [21], Combined Annotation Dependent Depletion (CADD) v1.7 [22], Rare Exome Variant Ensemble Learner (REVEL) (https://sites.google.com/site/revelgenomics/, accessed on 11 March 2026) [23], AlphaMissense (AM) (https://github.com/google-deepmind/alphamissense, accessed on 11 March 2026) [24], and ClinVar (https://www.ncbi.nlm.nih.gov/clinvar/, accessed on 11 March 2026) [25] (Figure 3).

After variant annotation, only variants with read depth (DP) > 10 and genotype quality (GQ) ≥ 99 were retained. In the family WES dataset, only variants consistent with an autosomal dominant inheritance model were selected, whereas in the individual WES dataset (non-family samples), only heterozygous variants were retained. Variants predicted to have high functional impact, including stop-gain and frameshift variants, were excluded if their allele frequency exceeded 1% in the general population. In contrast, variants with lower predicted impact, such as missense variants, were excluded if their allele frequency exceeded 0.01% in the general population.

After population allele frequency filtering, in silico pathogenicity assessment was performed, and variants with CADD < 20, REVEL < 0.5, or AM < 0.564 were excluded. Variants classified as benign or likely benign in ClinVar were also removed. A CADD score of 20 represents the top 1% most deleterious variants in the human genome [22]. A REVEL score of 0.5 is the PP3 supporting evidence threshold recommended by the ClinGen Sequence Variant Interpretation Working Group [26]. An AM score of 0.564 is the likely pathogenic cutoff proposed in the original AlphaMissense study [24].

Subsequently, only coding and splice-site variants with the potential to alter protein structure or function were retained. Final candidate variants were prioritized if they were located in genes belonging to hEDS-related pathways, including the hyaluronan (HA)–extracellular matrix (HA-ECM) axis (*CD44*, *ITIH* family, *HMMR*, and *STAB2*), extracellular matrix (ECM) remodeling (*ADAM*, matrix metalloproteinase [*MMP*], *ADAMTS*, and tissue inhibitor of metalloproteinases [*TIMP*] family genes), ECM structural components (e.g., *COL28A1*, *HSPG2*, *HMCN1*, *EMILIN2*, and *VWA* domain-containing genes), cell–matrix adhesion and mechanotransduction (e.g., integrin, FAK, Piezo, ephrin, and claudin-related genes), cytoskeleton regulation (e.g., spectrin, RhoGEF, actin dynamics, and PDZ-LIM-related genes), transforming growth factor-β (TGF-β)/bone morphogenetic protein (BMP)/Wnt signaling (e.g., *SOX9*, *KCP*, and *RSPO4*), collagen processing and endoplasmic reticulum (ER) quality control (e.g., *SEC23A*, *POMT1*, *DNAJB9*, and *EDEM2*), complement/immune/mast cell pathways (e.g., gasdermin, *FCRL4*, *IL-1*, *IL-36*, and *SIGIRR*-related genes), vascular/autonomic/ion channel pathways (e.g., *ANGPT2*, *SCN* family, *KCNH6*, and *TRPV6*), and muscle/cardiac/skeletal/wound-healing pathways (e.g., *TTN*, *CMYA5*, *ALPL*, *SLC26A2*, and *PROM1*) (Figure 3, and Supplementary File S3: Supplementary Tables S2–S4). These pathways were selected based on prior publications [27,28] addressing ECM organization, mechanotransduction, and connective tissue integrity in hEDS pathophysiology. The overall filtering workflow was encapsulated in a program named Autosomal-Dominant-Inherited-finder (ADIF) and is publicly available in our GitHub repository (ADIF v1.0.0, https://github.com/minjoocho215/ADIF_v1, accessed on 11 March 2026) for transparent and reproducible use.

### 2.5. Manual Anterior Drawer Test

The manual ADT was performed with the participant in a seated or supine position, as previously described by van Dijk et al. [15]. With the knee flexed and the lower leg supported, the ankle was positioned in 10–15° of plantarflexion. The examiner stabilized the tibia with one hand while grasping the heel with the other, and an anterior force was applied to the foot until a firm endpoint was reached. The degree of anterior displacement of the talus relative to the tibia was used to assess ligamentous laxity, and all cases were classified into three grades based on the test findings: grade I, stable joint; grade II, partial instability; and grade III, complete instability, which may be accompanied by a dimple sign [15,16].

### 2.6. Stress Ultrasound of Ankle

**Figure 4:**
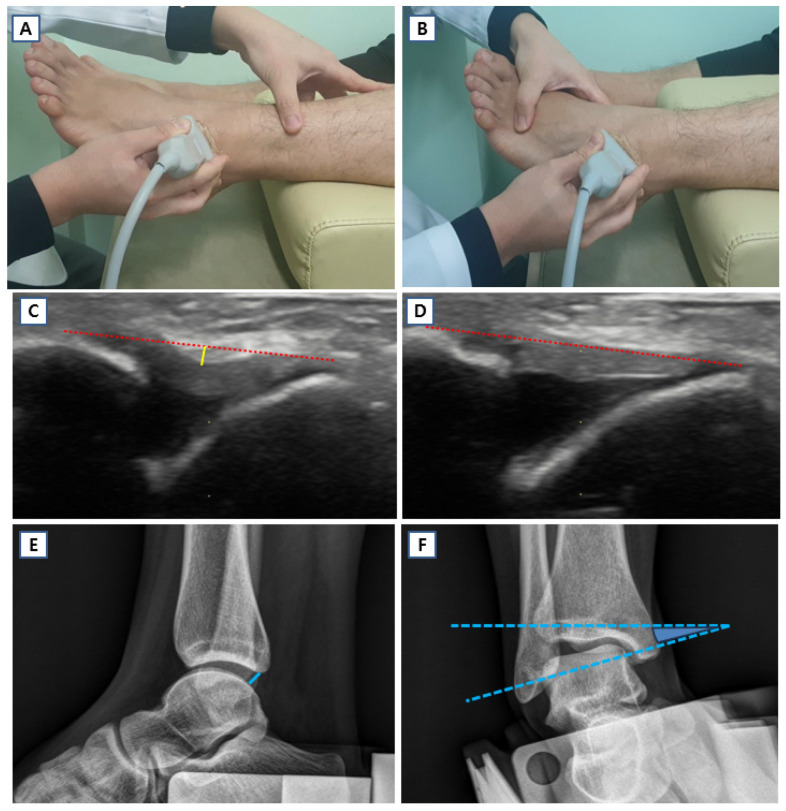
Stress ultrasonography and stress radiography of the ankle. Ultrasound images of the anterior talofibular ligament (ATFL) were obtained in (**A**) the resting position and (**B**) during maximal plantar flexion and inversion stress. Representative images from a 21-year-old dancer with a Beighton score of 7 are shown in (**C**) the resting position and (**D**) during stress. ATFL length (dotted red line) was defined as the linear distance between the talar and fibular attachment sites, and ATFL height (yellow line) was measured as the maximum perpendicular distance from the length line to the superficial border of the ATFL, representing ligament laxity. For stress radiography, (**E**) anterior drawer stress and (**F**) inversion stress were applied using a force of 150 N. Anterior talar translation (blue line) was measured as the distance between the posterior lip of the tibia and the nearest articular surface of the talus, and talar tilt was defined as the angle (blue angle) between reference lines drawn along the talar dome and the tibial plafond.

Resting and stress ultrasonography were performed to evaluate the anterior talofibular ligament (ATFL) in terms of its length and height, with height representing ligament laxity, following a previously established protocol [3]. In the previous study, this ultrasonographic protocol demonstrated good intra- and inter-observer reliability, with intraclass correlation coefficients exceeding 0.75 [3]. Imaging was conducted in the longitudinal plane using a z.one ultra ultrasound system equipped with a 12 MHz linear probe (ZONARE Medical Systems Inc., San Jose, CA, USA). Participants were positioned supine with the examined leg supported on a pillow, and the probe was aligned over the ATFL according to anatomical landmarks described by Matsui et al. [29]. Resting images were obtained with the ankle in mild plantar flexion (10–20°). Stress images were acquired by applying maximal plantar flexion and inversion force until full tension of the ATFL was observed on ultrasound. Ligament length was defined as the straight distance between the talar and fibular attachment sites, whereas ligament height was measured as the maximum perpendicular distance from this length line to the superficial border of the ATFL. Differences in ATFL parameters between resting and stress conditions, as well as the stress-to-rest ratio of ATFL length, were also analyzed (Figure 4) [16].

### 2.7. Stress Ankle X-Ray

Mechanical ankle stability was evaluated using stress radiographs obtained with a Telos device (Telos GmbH, Marburg, Germany) [16]. Anterior drawer and inversion stress force of 150 N were applied uniformly to all participants. Measurements included anterior talar translation and the talar tilt angle. Anterior talar translation was defined as the distance between the posterior lip of the tibia and the closest articular surface of the talus, while talar tilt was calculated as the angle between reference lines drawn along the talar dome and the tibial plafond. All radiographic measurements were performed digitally using picture archiving and communication system software (INFINITT PACS M6, INFINITT Healthcare, Seoul, Republic of Korea) (Figure 4).

### 2.8. Statistical Analysis

The statistical analysis was conducted by the study’s main author (J.-Y.K.), who has formal training in statistics. A priori power analysis was performed using G*Power (version 3.1.9.2). Assuming an alpha level of 0.05 and statistical power of 80%, the analysis determined that at least 20 participants per group would be necessary to detect a significant difference. Statistical analyses for comparing manual ADT grades, ultrasound parameters, and stress radiographic findings between the two groups were performed using SPSS version 22.0 (IBM Corp., Armonk, NY, USA). Data normality was assessed using the Shapiro–Wilk test. The Mann–Whitney U test was used for group comparisons. To control for the inflation of type I error arising from multiple imaging parameter comparisons, the Benjamini–Hochberg false discovery rate (FDR) correction was applied (hereafter denoted as *p*-adj.). Statistical significance was set at *p*-adj. < 0.05.

## 3. Results

### 3.1. Baseline Characteristics of the hEDS Cohort and Family Members

**Figure 5:**
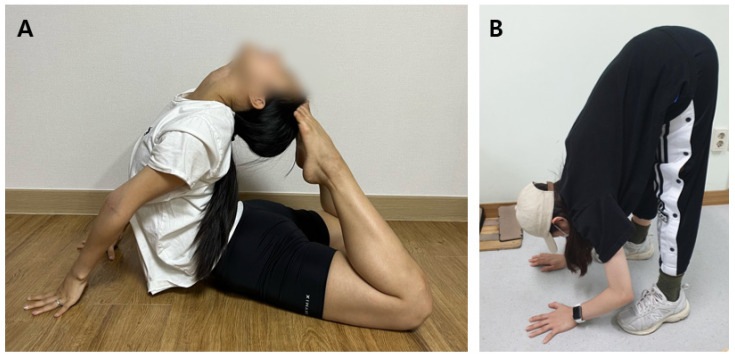
Representative images of patients with hEDS showing severe joint laxity. (**A**) Hyperextension of the lumbar spine. (**B**) Forward flexion of the trunk with the knees fully extended.

Through a public recruitment announcement at the foot and ankle clinic of Konyang University Hospital between June 2020 and June 2021, a total of 60 individuals were initially enrolled in this study (Figure 1). All recruited participants were between 20 and 40 years of age and were able to undergo evaluation including the Beighton score, manual ADT, stress ultrasonography, and stress radiography. After assessment of the Beighton score and clinical history, 30 individuals who did not meet the clinical diagnostic criteria for hEDS were excluded. In addition, 5 individuals with a history of ankle fracture or disease and 3 individuals with previous ankle surgery were excluded. Ultimately, 22 participants were included in the hEDS cohort (Table 2). All 22 participants had a family history of joint hypermobility and exhibited musculoskeletal complications. The cohort consisted of 20 females and 2 males, with a mean age of 21.86 years (range, 19–26 years). Seventeen participants were majoring in dance, including ballet, modern dance, or Korean dance, and both male participants were dance majors. The mean Beighton score was 8.5 out of 9; specifically, 16 participants scored 9, 2 scored 8, 3 scored 7, and 1 scored 5. Both male participants had a Beighton score of 9. Among musculoskeletal complications, knee pain was the most frequent finding (*n* = 13), followed by wrist pain (*n* = 7) and hip pain (*n* = 6). Regarding ankle symptoms (21 out of 22 participants, 95.5%), lateral ankle laxity was the most common finding (*n* = 15). Signs to joint laxity were present in 19 of 22 participants (86.4%), among which full extension of the hip was the most frequent finding (*n* = 14), followed by hyperextension of the lumbar spine (*n* = 3), external rotation of the shoulder (*n* = 2) (Supplementary File S2: Supplementary Video S1), and hyperflexion of the wrist (*n* = 1) (Figure 5).

In addition, for WES-based investigation of the genetic causes of hEDS, a three-generation Korean family comprising 6 individuals was included (Figure 1 and Supplementary File S3: Supplementary Table S1). Family members were diagnosed using the same criteria applied to the hEDS cohort. Of the 6 individuals, 4 were diagnosed with hEDS and 2 were unaffected spouses. The mean age of all 6 family members was 40.5 years, whereas the mean age of the 4 affected individuals was 36 years (range, 8–64 years). Among the affected family members, 3 had a Beighton score of 9 and 1 had a score of 8. For musculoskeletal complications, wrist pain was the most common finding (*n* = 3), followed by knee pain (*n* = 2). Regarding ankle symptoms, lateral ankle laxity was the most frequent finding in the second and third generations (*n* = 2), followed by ankle pain (*n* = 1).

### 3.2. Genetic Causes Identified by WES in a Three-Generation Family

**Figure 6:**
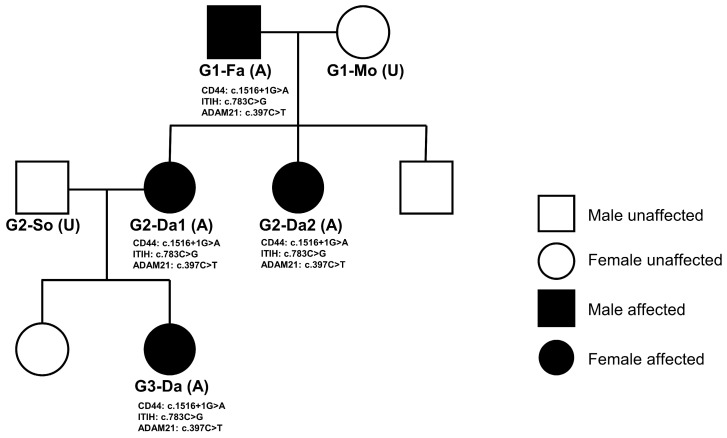
Pedigree of the three-generation Korean family with hEDS. Squares indicate males and circles indicate females. Filled symbols represent affected individuals with hEDS and are labeled as (A), whereas unfilled symbols represent unaffected individuals and are labeled as (U). G1, G2, and G3 denote the first, second, and third generations, respectively. Fa indicates father, Mo mother, Da daughter, and So son-in-law. The shared variants and corresponding genes identified across the affected family members are shown below each affected individual.

WES was performed to identify genetic causes of hEDS in a three-generation Korean family. The family was composed of 4 affected individuals with hEDS and 2 unaffected spouses (Figure 6). In this family-based dataset, a total of 1,436,367 initial germline variants corresponding to 28,696 genes were identified (Figure 3). To retain high-quality variants, only variants with a read depth of at least 10 and genotype confidence greater than 99.9999% were selected, resulting in 42,543 variants across 14,388 genes. To identify variants segregating across generations, only variants consistent with an autosomal dominant inheritance model were retained, yielding 256 variants in 233 genes. Because variants observed in the general population were considered more likely to represent common polymorphisms than disease-associated functional variants, additional filtering based on population allele frequency was applied. High-impact variants, including variants predicted to disrupt protein function such as stop-gained and frameshift variants, were excluded if their allele frequency exceeded 1% in the general population, whereas other variants were excluded if their allele frequency exceeded 0.01%. In addition, variants predicted to have low pathogenicity by in silico analyses were removed (see Methods), resulting in 32 variants in 24 genes. When only coding or splice-site variants with potential functional consequences were considered, 5 variants in 5 genes remained. The candidate variants were further restricted to genes involved in hEDS-related pathways, including the HA-ECM axis and ECM remodeling. Ultimately, 3 variants in 3 genes were identified as shared across the pedigree: *CD44*, *ITIH2*, and *ADAM21* (Table 3 and Figure 6).

All four affected family members harbored all three variants, whereas the two unaffected spouses carried none, demonstrating complete co-segregation of these variants with the hEDS phenotype across three generations (Table 4). Consistent with this segregation pattern, the affected individuals exhibited high Beighton scores (≥8) and musculoskeletal complications, with ankle symptoms (lateral ankle instability or ankle pain) observed in the second and third generations, whereas the two unaffected spouses showed Beighton scores below the diagnostic threshold (4 each) and no ankle involvement.

*CD44*, which belongs to the HA-ECM axis, is located on chromosome 11 at position 35,208,207. The identified variant, c.1516 + 1G > A, is a splice-donor lost variant in which the first intronic nucleotide immediately following coding position 1516 is changed from G to A. Such a variant may disrupt normal splicing by causing exon skipping, intron retention, or activation of a cryptic splice site, potentially resulting in reading-frame alteration, abnormal mRNA processing, or production of an abnormal protein. *ITIH2*, which also belongs to the HA-ECM axis, is located on chromosome 10 at position 7,721,693. The identified variant, c.783C > G (p.Cys261Trp), is a missense variant in which cysteine at amino acid position 261 is replaced by tryptophan, potentially affecting protein structure or functional interactions through changes in physicochemical properties. *ADAM21*, which belongs to the ECM remodeling pathway, is located on chromosome 14 at position 70,457,896. The identified variant, c.397C > T (p.Arg133Ter), is a stop-gained variant that introduces a premature termination codon at amino acid position 133, which may result in protein truncation or degradation of the transcript through nonsense-mediated decay and is therefore likely to be associated with loss of normal protein function. These three genes and their corresponding variants were identified in common across all four affected individuals within the pedigree (G1-Fa, G2-Da1, G2-Da2, and G3-Da) (Supplementary File S3: Supplementary Table S2).

### 3.3. Candidate Genes Associated with Genetic Causes Identified by WES in 22 hEDS Patients

To identify genetic causes underlying hEDS in 22 patients with a family history of joint hypermobility and musculoskeletal complications, WES was performed. A total of 1,148,670 initial germline variants corresponding to 28,277 genes were identified across the 22 patients (Figure 3), with an average of 74,883 variants across 12,857 genes per patient. Subsequently, the same filtering strategy applied to the three-generation Korean family WES dataset was implemented. First, variants with a read depth ≥10 and genotype quality ≥99.9999% were retained, yielding 89,604 variants across 17,967 genes (average: 8764 variants across 3121 genes per patient). Second, only heterozygous variants consistent with an autosomal dominant inheritance model were selected, resulting in 88,955 variants across 17,936 genes (average: 8575 variants across 3070 genes per patient). Third, variants reported in the general population were excluded based on allele frequency thresholds, reducing the dataset to 17,437 variants across 4785 genes (average: 3042 variants across 378 genes per patient). Fourth, variants predicted to have low pathogenicity based on in silico analyses were further removed, leaving 16,344 variants across 4186 genes (average: 2917 variants across 338 genes per patient). Finally, by restricting to coding and splice-site variants within hEDS-related pathways, 114 variants implicating 103 candidate genes were identified (average: 5.2 variants across 4.7 genes per patient). As these genes were derived from individual patient WES analyses without familial co-segregation, rather than from pedigree-based WES of the three-generation Korean family, they were considered candidate genes associated with the genetic causes of hEDS rather than definitive causal genes.

Of the 114 variants identified across the 103 candidate genes, 69 were single-nucleotide variants, while insertion and deletion variants comprising two or more nucleotides accounted for 22 and 23 variants, respectively (Supplementary File S3: Supplementary Table S4). The most frequent variant type was the frameshift variant (*n* = 31), which results from insertions or deletions within the protein-coding region that generate aberrant amino acid sequences or lead to premature truncation. This was followed by missense variants (*n* = 27), in which single-nucleotide substitutions result in amino acid changes that may alter protein function, and stop-gained variants (*n* = 24), which introduce premature termination codons leading to early termination of protein synthesis. Additionally, splice-acceptor (*n* = 12) and splice-donor variants (*n* = 8), which occur at exon–intron boundary recognition sites and may disrupt normal RNA splicing, were also identified. From the perspective of the 103 candidate genes, the most frequently implicated hEDS-related pathways were cytoskeleton-related pathways (*n* = 15), followed by vascular/autonomic/ion channel pathways (*n* = 14), cell–matrix adhesion/ECM remodeling, mechanotransduction, and complement/immune/mast cell pathways (*n* = 12 each), and ECM structural pathways (*n* = 11) (see Supplementary File S3: Supplementary Table S4 for details). Among the 103 candidate genes, seven genes harbored two or more variants: *FCRL4*, *CAPN9*, *TRPV6*, *CGREF1*, *KCP*, *XIRP2*, and *FCGBP*. The pathways implicated by these recurrently mutated genes—ECM remodeling, TGF-β/BMP signaling, ECM structural components, cytoskeleton regulation, and ion channel function—represent core biological pathways underlying hEDS pathophysiology [27,28].

Across all 22 patients, each individual harbored variants in genes spanning at least two core hEDS-related pathways with potential functional impact. Notably, in patient 17, a splice-donor variant (c.1119 + 1G > A) was identified in *COL11A2*, a gene known to be associated with EDS and Stickler syndrome. In patient 6, a frameshift variant (c.1559del) was identified in *ALPL*, a gene associated with skeletal and connective tissue disorders; a frameshift variant (c.1662del) in *ITIH6*, which is involved in HA-ECM stabilization; and a stop-gained variant (c.76A > T) in *RLN1*, which is implicated in collagen degradation and ECM remodeling. In patient 1, a splice-acceptor variant (c.1635-1G > T) was identified in *ADAM32*, a member of the *ADAM* metalloproteinase family involved in ECM degradation and remodeling; an inframe insertion variant (c.1732_1743dup) in *MMP24*, a matrix metalloproteinase; and stop-gained variants in *DNAJB9* (c.247C > T) and *EDEM2* (c.1090C > T), both belonging to the endoplasmic reticulum quality control pathway associated with collagen secretion (Table 5). Similarly, in the remaining patients, genes harboring variants with potential relevance to the hEDS phenotype were consistently identified, supporting the relevance of the 103 candidate genes as potential genetic contributors to hEDS (Supplementary File S3: Supplementary Table S3).

Among the 22 patients, only patient 2 was found to carry a variant in one of the three genes (*CD44*, *ITIH2*, and *ADAM21*) identified as genetic causes in the three-generation Korean family (Table 3 and Table 4). Specifically, patient 2 harbored a single splice-acceptor variant (c.2694-1G > A) in *ITIH2*. From a pathway perspective, a total of 17 patients, including patient 2, carried variants in genes belonging to the same pathways as these three family-derived causative genes—namely, HA-ECM, ECM remodeling, and cell adhesion. Among these, the ECM remodeling pathway was the most frequently represented, followed by cell adhesion and HA-ECM pathways. In the remaining five patients in whom no variants were identified in the family-derived causative genes or their associated pathways, variants were detected in *SCN3A*, *SCN9A*, *TRPV6*, *KCP*, and *COL28A1*, implicating the ion channel, ECM structural, and cytoskeleton pathways (Table 5). Taken together, the three causative genes identified from the family-based analysis were directly or indirectly recapitulated in 17 of the 22 individual patients, either through variants in the same genes or in genes belonging to the same pathways.

### 3.4. Ankle Instability Assessment

**Figure 7:**
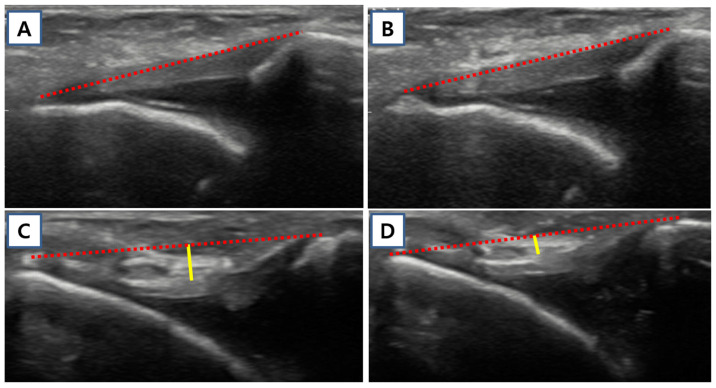
Representative stress ultrasonography images of the anterior talofibular ligament (ATFL). (**A**,**B**) A 20-year-old woman from the control group in the resting (**A**) and stress (**B**) positions. (**C**,**D**) A 26-year-old woman with hEDS in the resting (**C**) and stress (**D**) positions. The red dotted line indicates ATFL length and the yellow line indicates ATFL height, representing the degree of ligament laxity.

Manual ADT, stress ultrasonography, and stress radiography were performed in the hEDS cohort to evaluate ankle status. For comparative analysis, results were compared with those of a non-GJH control group (*n* = 24) derived from our previous cross-sectional cohort study [3], in which healthy young participants (age 20–40 years) without GJH (Beighton score < 5) were evaluated using the same ultrasonographic protocol. Manual ADT grades were significantly higher in the hEDS group than in the control group (mean 2.59 vs. 1.67; *p*-adj. < 0.001), indicating a greater degree of ligamentous laxity in patients with hEDS (Table 6). Stress ultrasonography demonstrated that resting ATFL length (mean 19.55 mm vs. 18.45 mm; *p*-adj. = 0.013) and resting ATFL height (mean 0.95 mm vs. 0.21 mm; *p*-adj. = 0.020) were both significantly greater in the hEDS group than in the control group, while stress ATFL height did not differ significantly between groups (mean 0.18 mm vs. 0.10 mm; *p*-adj. = 0.304). Under stress conditions, ATFL length remained significantly greater in the hEDS group (mean 21.18 mm vs. 19.63 mm; *p*-adj. = 0.010). The between-condition differences in ATFL length (mean 1.64 mm vs. 1.18 mm; *p*-adj. = 0.034) and height (mean 0.77 mm vs. 0.11 mm; *p*-adj. = 0.012) were both significantly greater in the hEDS group, indicating greater dynamic ligamentous deformation under mechanical loading, and the stress-to-resting ATFL length ratio was likewise significantly higher in the hEDS group (mean 1.08 vs. 1.07; *p*-adj. = 0.043) (Figure 7). These findings are consistent with our previous observation that the ATFL tends to exhibit a tight, linear morphology with relatively static motion in patients with low Beighton scores, whereas those with high Beighton scores demonstrate a loose, wavy pattern at rest and more dynamic flattening and stretching motion under stress [3]. Stress ankle radiography further corroborated these results, with anterior talar translation (mean 5.86 mm vs. 3.50 mm; *p*-adj. < 0.001) and talar tilt (mean 7.68° vs. 4.29°; *p*-adj. = 0.012) both significantly greater in the hEDS group. Taken together, stress ultrasonography and stress radiography successfully characterized the ankle status of patients with hEDS, consistently demonstrating significantly greater ankle laxity across the majority of measured parameters compared with the non-GJH control group (Table 6).

## 4. Discussion

The most important finding of the present study was the identification of candidate genetic variants associated with hEDS through WES. In parallel, this study also showed that patients with hEDS exhibited significantly greater ankle instability than controls, as reflected by manual ADT grade, ultrasonographic ATFL parameters, and stress radiographic measurements.

As noted above, disorders classified as EDS are divided into 13 subtypes according to their predominant clinical features [1] (Table 1), yet they share a common set of core manifestations, including joint hypermobility, skin hyperextensibility, tissue fragility, and easy bruising. Among these subtypes, hEDS is the most common and is characterized mainly by chronic pain and joint laxity. However, unlike the other subtypes, its genetic basis remains unresolved. Accordingly, extensive efforts have been made to define its molecular background, including the identification of *LZTS1* in a large Belgian family [30] and transcriptomic analyses [31], but the precise pathogenic mechanism remains unclear.

Joint hypermobility is not a clinical feature unique to EDS, but rather a shared characteristic across a broad spectrum of disorders, including HCTDs [5,6]. Marfan syndrome (MS) and Loeys–Dietz syndrome are representative examples [32], and both show substantial clinical overlap with EDS in cardiovascular, skeletal, craniofacial, ocular, and cutaneous manifestations. Although MS is genetically defined by mutations in *FBN1*, the clinical features it shares with several EDS subtypes, including aortic aneurysm, scoliosis, and retinal detachment, suggest that at least part of the underlying genetic factors and pathogenic mechanisms may overlap. Indeed, cases of cEDS have been reported in which large genomic duplications involving *COL5A1* coexist with skeletal features resembling MS [27], and *COL5A1* has also been implicated in congenital scoliosis through altered methylation [33] and in aortic dissection through regulation of TGF-β signaling [34]. This concept of genetic and functional convergence provides an important framework for interpreting the findings of the present study.

**Figure 8:**
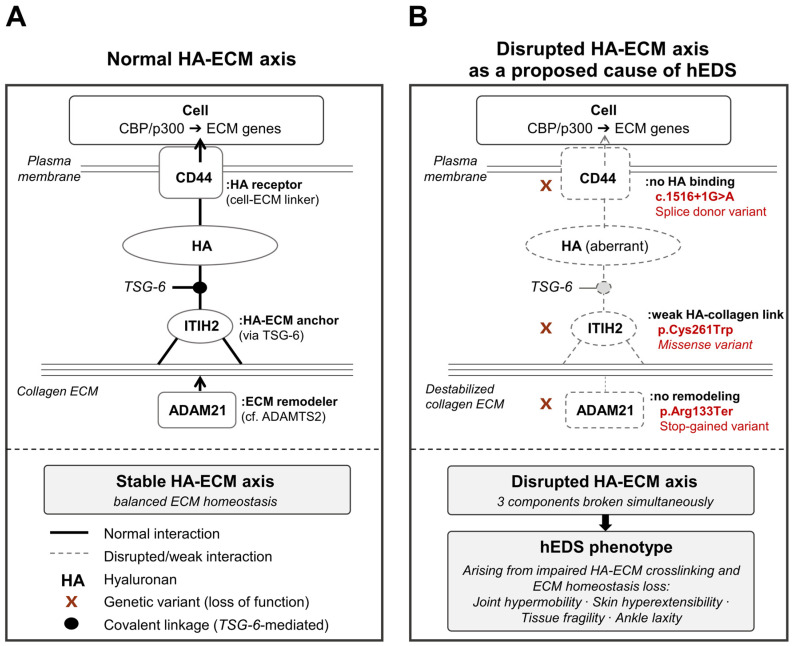
Schematic model of the HA-ECM axis as a proposed mechanism underlying the hEDS phenotype in the three-generation Korean family. (**A**) Normal HA-ECM axis. *CD44* (HA receptor at the plasma membrane), *ITIH2* (HA-ECM anchor, covalently linked to HA through TSG-6; filled circle), and *ADAM21* (ECM remodeler, related to *ADAMTS2*) coordinately maintain ECM homeostasis. Arrows indicate the direction of intracellular signaling (*CD44* → CBP/p300 → ECM gene expression) and enzymatic action (*ADAM21* → collagen). (**B**) Disrupted HA-ECM axis as a proposed cause of hEDS. The three variants identified in the family—*CD44* c.1516 + 1G > A (splice donor), *ITIH2* c.783C > G p.Cys261Trp (missense), and *ADAM21* c.397C > T p.Arg133Ter (stop-gained)—are predicted to simultaneously disrupt HA binding, HA–collagen anchoring, and ECM remodeling. Adjacent annotations indicate the predicted loss-of-function consequence, the genetic variant, and the variant class for each gene. X marks denote sites of loss of function; dashed lines indicate aberrant or weakened interactions. The combined dysfunction is proposed to destabilize the collagen-rich ECM, giving rise to the hEDS phenotype.

In the three-generation Korean family, WES identified three variants shared across all affected individuals: *CD44* (c.1516 + 1G > A, splice-donor variant), *ITIH2* (c.783C > G, p.Cys261Trp, missense variant), and *ADAM21* (c.397C > T, p.Arg133Ter, stop-gained) (Table 3, Figure 6, Supplementary File S3: Supplementary Tables S1 and S2). Of particular note, both *CD44* and *ITIH2* belong to the HA-ECM axis, raising the possibility that simultaneous disruption of both ends of this pathway may have contributed to the phenotype observed in this family. Under normal conditions, *ITIH2* is covalently linked to hyaluronan via TSG-6 (TNF-stimulated gene 6) and functions as an anchor protein that physically stabilizes HA within collagen-rich ECM while contributing to the localization, synthesis, and degradation of HA [27] (Figure 8A). *CD44*, in turn, serves as a major cell-surface receptor for HA, recognizes and binds HA through its N-terminal domain [28], and may further regulate ECM-related gene expression through intracellular signaling and transcriptional mechanisms involving CBP/p300 [28]. In this family, the *ITIH2* p.Cys261Trp variant is predicted to impair protein structure and HA-related binding function (REVEL 0.646, AM 0.91), which could weaken HA-collagen crosslinking and destabilize the ECM scaffold (Figure 8B). The *CD44* c.1516 + 1G > A variant affects the canonical +1 splice site and is therefore expected to induce exon skipping or aberrant splicing, resulting in loss of normal HA binding and downstream ECM regulatory function. In other words, both the protein anchoring HA to the ECM and the receptor sensing HA at the cellular level may be impaired, suggesting impaired integrity of the HA-ECM axis. The *ADAM21* p.Arg133Ter variant may have additionally contributed through abnormal ECM remodeling (Figure 8B). The *ADAM* family belongs to the same metzincin metalloprotease superfamily as *ADAMTS2*, an established EDS-related gene, and plays an important role in ECM substrate processing [1]. Taken together, the joint hypermobility, skin hyperextensibility, tissue fragility, and ankle laxity observed in this family can be interpreted as reflecting disruption of ECM homeostasis caused by dual impairment of the HA-ECM axis. In this respect, our findings provide molecular support for the recently proposed shift in perspective that the core pathology of hEDS and hypermobility spectrum disorder (HSD) may lie less in primary structural defects of collagen fibrils than in disturbed cell–matrix adhesion [27,28,35].

In the 22 individual patients, variants involving at least two core hEDS-related pathways were identified in every patient, yielding a total of 114 variants across 103 candidate genes (Table 5, Supplementary File S3: Supplementary Tables S3 and S4). The most frequently represented pathways were cytoskeleton regulation (*n* = 15), vascular/autonomic/ion channel pathways (*n* = 14), cell–matrix adhesion, ECM remodeling, mechanotransduction, and complement/immune/mast cell pathways (*n* = 12 each), followed by ECM structural components (*n* = 11). Within the ECM remodeling pathway, variants were identified in multiple metalloproteinase-related genes, including *MMP8* (patient 10), *MMP24* (patient 1), *MMP7* (patient 21), *ADAM32*, *ADAM7*, *ADAM33*, *ADAMTS7P1*, and *TIMP1*. *MMP8* directly degrades type I, II, and III collagen, whereas *TIMP1* inhibits *MMP* activity and protects the ECM; thus, concurrent disruption of these genes may indicate dysregulation of ECM turnover, reflecting imbalance of the *MMP*–*TIMP* axis. In the collagen processing and secretion pathway, variants were identified in *SEC23A* (patient 18) and in *DNAJB9* and *EDEM2* (patient 1). *SEC23A* is a core component of the coat protein complex II required for ER-to-Golgi transport of collagen, and pathogenic variants in *SEC23A* are known to cause collagen secretion defects in cranio-lenticulo-sutural dysplasia [36]. In the TGF-β/BMP signaling pathway, variants were identified in *KCP* (patients 4 and 11) and *SOX9* (patient 19). *KCP* enhances BMP signaling while suppressing TGF-β signaling [37], and the presence of the same loss-of-function variant (c.169C > T, p.Arg57Ter) in two unrelated patients supports the possibility that dysregulation of this pathway may be involved in hEDS, consistent with previous reports [27]. *SOX9* is a master transcription factor of chondrogenesis and directly regulates collagen genes including *COL2A1* and *COL11A2* [38,39]; thus, the inframe insertion identified in patient 19 may contribute to altered regulation of collagen-related pathways. In the cytoskeleton pathway, *XIRP2* was identified in two unrelated patients (patients 16 and 19) with distinct stop-gained variants. In the ion channel pathway, variants were identified in *SCN9A* (patient 22), *SCN5A* (patient 5), *SCN3A* (patient 13), and *TRPV6* (patient 17). Direct collagen-related variants included a *COL11A2* splice-donor variant (c.1119 + 1G > A, Likely Pathogenic) in patient 17, a *COL28A1* missense variant in patient 16, and an *ALPL* frameshift variant (c.1559del, ClinVar Pathogenic) in patient 6. Notably, genes belonging to the same pathways as the three family-identified genes were found in 17 of the 22 patients (77.3%), supporting a broad involvement of the HA-ECM axis and related pathways across the individual hEDS cohort.

Patients 11 and 22 showed the weakest direct association with hEDS-related ECM pathways (Table 5). Patient 11 did not harbor variants in ECM structural or collagen-related genes; however, the *KCP* stop-gained variant (c.169C > T) is a loss-of-function alteration affecting the TGF-β/BMP pathway and may still have sufficient biological plausibility to contribute to hEDS [37], in agreement with previous reports [27]. The accompanying SPTBN4 missense variant (c.5772G > C) was predicted to be pathogenic by AM and may additionally impair the mechanical buffering role of the cytoskeleton–ECM interface (Supplementary File S3: Supplementary Table S4). Patient 22 also lacked direct ECM or collagen-related variants, but carried a missense variant in *SCN9A* (c.167G > A), a gene that encodes Nav1.7 and plays a central role in chronic pain and autonomic dysfunction. Dysfunction of *SCN9A* has been associated with small fiber neuropathy and widespread allodynia, both of which are frequently reported in hEDS [32]. In addition, the *MYOM2* splice-acceptor variant (c.3695-2A > G) may affect myomesin-2, a structural protein involved in sarcomeric M-band stability, and may therefore contribute to muscle hypermobility and susceptibility to fatigue [32]. From this perspective, patient 22 may be understood within the context of neuromuscular–connective tissue axis dysfunction rather than primary ECM structural abnormality [1]. Overall, these findings support the interpretation of hEDS as a complex multifactorial disorder arising from the cumulative effects of variants across interconnected biological pathways, including ECM organization, cell–matrix adhesion, cytoskeletal regulation, TGF-β/BMP signaling, collagen processing and secretion, and ion channel function, rather than from a single causative gene. This integrated interpretation offers a coherent explanation for both the high prevalence of hypermobility-associated disorders and the difficulty of identifying a single molecular diagnostic marker for hEDS [27,28].

The second aim of the present study was to objectively evaluate ankle status in patients with hEDS using stress ultrasonography and stress radiography (Table 6, Figure 4 and Figure 7). Compared with the control group, the hEDS group showed significantly greater ankle instability across the majority of measured parameters, including manual ADT grade, ultrasonographic ATFL parameters, and stress radiographic measurements. On ultrasonography, resting ATFL length and height were significantly greater in the hEDS group, and stress ATFL length was also significantly increased. In addition, the differences in ATFL length and height between resting and stress conditions were both larger in the hEDS group, indicating greater dynamic ligament deformation under loading. The mean ATFL length ratio was significantly higher in the hEDS group (1.08) than in the control group (1.07). Studies by Yokoe et al. [40,41], conducted separately in young women and men, also demonstrated significant differences in the ATFL ratio between individuals with and without generalized joint laxity, thereby supporting the findings of the present study. These findings are consistent with our previous observation that patients with low Beighton scores tend to show a relatively tight and linear ATFL morphology, whereas those with high Beighton scores show a looser and more wavy ligament pattern at rest [3]. Stress radiography also demonstrated significantly greater anterior talar translation and talar tilt in the hEDS group. Taken together, these findings suggest that ankle instability is a clinically meaningful feature of hEDS that is not adequately reflected in the current Beighton score [14,17], and that ankle-specific assessment using stress ultrasonography and stress radiography provides a clinically valuable objective complement to conventional physical examination. To our knowledge, this is the first study to investigate both the genetic background of hEDS and ankle status using stress ultrasonography and stress radiography, providing new evidence for both the molecular basis of hEDS and the clinical utility of ankle-specific evaluation.

This study has several limitations. First, hEDS is highly genetically heterogeneous [32] and a single pedigree dataset cannot fully explain its genetic causes. Accordingly, *CD44*, *ITIH2*, and *ADAM21* should be regarded as representing only a small subset of possible hEDS-related genes (Table 3). Furthermore, the WES-based approach captures only protein-coding variants and therefore does not assess regulatory variants in non-coding regions or epigenetic factors that may contribute to hEDS pathogenesis. Nevertheless, pedigree-based co-segregation analysis across three generations of affected and unaffected individuals provides important support for the reliability of these findings (Figure 6) [1]. Even so, definitive establishment of their causal role in hEDS pathogenesis would require further experimental validation through functional studies. Second, the 22-patient hEDS cohort was not family-based, and co-segregation analysis was therefore not possible. As a result, high-penetrance variants segregating across pedigrees could not be identified, which limits definitive assignment of causative genes. Even so, the use of strict eligibility criteria based on the 2017 international classification [1] (Figure 1), inclusion only of patients with a family history, and rigorous multi-step variant filtering support the reliability of the candidate variants and genes presented in this study. Third, the sample size was relatively small, and a substantial proportion of the hEDS cohort consisted of professional dancers. Therefore, the potential effects of repetitive stretching and physical training on musculoskeletal ligament status and Beighton scores cannot be excluded, and these factors may have acted as confounders. Accordingly, caution is needed when generalizing these findings to the broader hEDS population. Fourth, potential confounders that may influence ultrasonographic ATFL findings, including sex, age, stretching habits, and dance training, were neither systematically evaluated nor adjusted for in the statistical analysis [3]. The small sample size and the marked imbalance in sex distribution within the hEDS group (20 females and 2 males) limited the feasibility of multivariable adjustment, and these factors should be considered when interpreting the findings. Fifth, the reliability of stress radiographic measurements was not assessed in this study. In addition, the control group was derived from our previous study [3]; however, all clinical examinations and ultrasound evaluations in both studies were performed by the same orthopedic surgeon using the same protocol, which helps mitigate limitations related to non-contemporaneous data collection. Sixth, the pathway-level interpretations presented in this study should be regarded as exploratory and hypothesis-generating, as they were derived from candidate gene assignments without direct functional or experimental validation. Although the identified variants implicate biologically relevant pathways, definitive establishment of their pathogenic relevance in hEDS will require further mechanistic studies.

## 5. Conclusions

Elucidating the genetic basis and ankle characteristics of hEDS is expected to provide a more structured foundation for the development of diagnostic and therapeutic strategies. In this study, WES identified candidate genetic variants associated with hEDS and provided molecular evidence supporting the interpretation of hEDS as a complex multifactorial disorder, in which multiple interconnected biological pathways collectively contribute to disease pathophysiology. In addition, objective evaluation of ankle status using stress ultrasonography and stress radiography identified ankle instability as a potentially important clinical feature in the diagnostic assessment of hEDS. Although this study provides preliminary evidence regarding both the molecular basis and clinical characteristics of hEDS through WES-based genetic analysis and objective assessment of ankle instability, further large-scale multicenter studies involving a greater number of patients with hEDS are warranted to more definitively establish its genetic basis and causation, and to clinically validate its association with ankle laxity.

## Figures and Tables

**Table 1:** Revised EDS ^a^ classification according to the 2017 international guidelines.

Clinical EDS Subtypes	Abbreviation	Inheritance Pattern	Genetic Basis	Protein
Classical EDS	cEDS	AD ^b^	Major: *COL5A1*, *COL5A2* Rare: *COL1A1* c.934C > T, p.(Arg312Cys)	Type V collagen Type I collagen
Classical-like EDS	clEDS	AR ^c^	TNXB	Tenascin XB
Cardiac-valvular	cvEDS	AR	*COL1A2* (biallelic mutations that lead to *COL1A2* nonsense-mediated decay and absence of pro α2(I) collagen chains)	Type I collagen
Vascular EDS	vEDS	AD	Major: *COL3A1* Rare: *COL1A1* c.934C > T, p.(Arg312Cys) c.1720C > T, p.(Arg574Cys) c.3227C > T, p.(Arg1093Cys)	Type III collagen Type I collagen
Hypermobile EDS	hEDS	AD	Unknown	Unknown
Arthrochalasia EDS	aEDS	AD	*COL1A1*, *COL1A2*	Type I collagen
Dermatosparaxis EDS	dEDS	AR	*ADAMTS2*	ADAMTS-2
Kyphoscoliotic EDS	kEDS	AR	*PLOD1* *FKBP14*	LH1 FKBP22
Brittle cornea syndrome	BCS	AR	*ZNF469* *PRDM5*	ZNF469 PRDM5
Spondylodysplastic EDS	spEDS	AR	*B4GALT7* *B3GALT6* *SLC39A13*	β4GalT7 β3GalT6 ZIP13
Musculocontractural EDS	mcEDS	AR	*CHST14* *DSE*	D4ST1 DSE
Myopathic EDS	mEDS	AD or AR	*COL12A1*	Type XII collagen
Periodontal EDS	pEDS	AD	*C1R* *C1S*	C1r C1s

^a^ EDS, Ehlers–Danlos syndromes; ^b^ AD, autosomal dominant; ^c^ AR, autosomal recessive.

**Table 2:** Baseline characteristics of the 22 participants included in the hEDS cohort.

Participant Number	Sex	Age	Dance Major	Beighton Score	Family History	Musculoskeletal Complications	Ankle Symptom	Events Related with Joint Laxity
1	F	20	−	8	+	Back/Wrist pain	Lateral ankle laxity	External rotation of shoulder
2	F	23	−	9	+	Wrist pain	Lateral ankle laxity	Full extension of hip
3	F	20	+	9	+	Hip pain	Lateral ankle laxity	Full extension of hip
4	F	23	+	9	+	Chronic shoulder dislocations (Postop. state), Wrist/Knee pain	Lateral ankle laxity	Full extension of hip
5	F	19	+	9	+	Back/Hip/Knee pain	Ankle pain	Full extension of hip
6	F	26	+	7	+	Knee pain	Lateral ankle laxity	Full extension of hip
7	F	22	+	9	+	Knee pain	Lateral ankle laxity	Full extension of hip
8	F	22	+	9	+	Knee pain	Prehallux syndrome	-
9	F	20	+	9	+	Shoulder pain	Ankle pain	External rotation of shoulder
10	F	21	+	9	+	Back/Knee pain	Lateral ankle laxity	Hyperextension of lumbar
11	F	23	+	9	+	Back/Knee pain (Anterior cruciate ligament rupture)	Ankle pain	-
12	F	23	+	9	+	Chronic shoulder dislocations, Knee/Wrist pain	Lateral ankle laxity	Hyperextension of lumbar
13	F	20	+	9	+	Hip/Knee pain	Ankle pain	Full extension of hip
14	F	22	+	9	+	Knee pain	Lateral ankle laxity	-
15	M	20	+	9	+	Hip pain	Lateral ankle laxity	Full extension of hip
16	F	25	+	9	+	Knee pain	Lateral ankle laxity	Hyperflexion of wrist
17	F	22	−	7	+	Shoulder/Wrist pain	Ankle pain	Full extension of hip
18	F	20	−	9	+	Wrist pain	Lateral ankle laxity	Full extension of hip
19	F	22	+	7	+	Shoulder/Knee pain	-	Full extension of hip
20	F	21	+	5	+	Wrist/Hip/Knee pain	Lateral ankle laxity	Full extension of hip
21	F	23	−	8	+	Hip pain	Lateral ankle laxity	Full extension of hip
22	M	24	+	9	+	Shoulder subluxation	Lateral ankle laxity	Full extension of hip, Hyperextension of lumbar

**Table 3:** Three candidate genes identified as genetic causes in the hEDS family dataset.

Gene	CHR	Position	HGVSc ^a^	HGVSp ^b^	Consequence	Pathway
*CD44*	11	35,208,207	c.1516 + 1G > A	Splice donor lost	Splice donor variant	HA-ECM ^c^ axis
*ITIH2*	10	7,721,693	c.783C > G	p.Cys261Trp	Missense variant	HA-ECM axis
*ADAM21*	14	70,457,896	c.397C > T	p.Arg133Ter	Stop gained	ECM ^d^ remodeling

^a^ HGVSc, Human Genome Variation Society coding sequence nomenclature; ^b^ HGVSp, Human Genome Variation Society protein sequence nomenclature; ^c^ HA-ECM, Hyaluronan–extracellular matrix; ^d^ ECM, Extracellular matrix.

**Table 4:** Co-segregation of *CD44*, *ITIH2*, and *ADAM21* variants with clinical features in the three-generation Korean hEDS family.

ID	Sex	Age	Beighton	FH ^a^	MS Complications	Ankle Symptom	*CD44* ^a^	*ITIH2* ^b^	*ADAM21* ^c^
G1-Fa (A)	M	64	8	+	Back/Neck pain	-	+	+	+
G1-Mo (U)	F	61	4	+	Knee pain	-	−	−	−
G2-So (U)	M	38	4	+	-	-	−	−	−
G2-Da1 (A)	F	38	9	+	Wrist/Knee pain	Lateral ankle instability	+	+	+
G2-Da2 (A)	F	34	9	+	Wrist/Knee pain	Ankle pain	+	+	+
G3-Da (A)	F	8	9	+	Wrist/Ankle pain	Lateral ankle instability	+	+	+

^a^ *CD44* c.1516 + 1G > A (splice-donor variant); ^b^ *ITIH2* c.783C > G, p.Cys261Trp (missense variant); ^c^ *ADAM21* c.397C > T, p.Arg133Ter (stop-gained variant). A, affected; U, unaffected; FH, family history of joint hypermobility; MS, musculoskeletal; +, present; −, absent.

**Table 5:** Candidate variants and genes for genetic causes identified by WES in 22 hEDS patients.

Participant Number	Target Variants	Target Genes	Target Genes Related to hEDS Pathways ^a^	Related hEDS Pathways
1	7	7	***ADAM32**, DNAJB9, EDEM2, FGFBP1, KCNH6, **MMP24**, SPTA1*	Cytoskeleton, ER quality control, Growth factor, Ion channel
2	4	4	*EMILIN2, **ITIH2**, NEBL, TRDN*	Cardiac, ECM structural, Muscle
3	5	5	***EFNB2**, FGD4, TECTA, TRIM72, VWA5B1*	Cytoskeleton, ECM structural, Membrane repair
4	4	4	***ASAP1**, KCP, SSH2, TICAM2*	Cytoskeleton, Immune, TGF-β/BMP
5	3	3	***NFASC***, *RSPO4, SCN5A*	Ion channel, Wnt
6	5	5	*ALPL, F7, GFAP, **ITIH6, RLN1***	Coagulation, Cytoskeleton, Skeletal
7	7	5	***CLDN1**, FCRL4, MEGF10, NOSTRIN, SHANK2*	Cytoskeleton, ECM structural, Immune, Vascular
8	8	7	*CGREF1, GSDME, IL36B, PCDHGA11, **PLA2R1**, PROM1, **THSD4***	ECM structural, Immune, Wound healing
9	8	8	***ADAM7**, AREG, **ATRN**, **CLDN2**, FCGBP, IL1A, **SEMA4A**, SLC26A2*	Growth factor, Immune, Skeletal
10	8	8	***ADAM33**, CHRNA3, COX7C, **MMP8**, MYH8, PCDHGB4, **PTPRM**, VWA3B*	ECM structural, Immune, Ion channel, Mitochondria, Muscle
11	2	2	*KCP, SPTBN4*	Cytoskeleton, TGF-β/BMP
12	6	6	*CMYA5, GSDMA, HMCN1, **PTPRQ**, SIGIRR, TCIRG1*	ECM structural, Immune, Muscle
13	4	4	*PDGFRL, PKHD1L1, SCN3A, USH2A*	ECM structural, Growth factor, Ion channel
14	7	7	***ADAMTS7P1**, KCNQ4, MYL6, OSMR, **TIMP1**, TUBA3C, WIPF3*	Cytokine, Cytoskeleton, Ion channel
15	6	6	*ANGPT2, ANK2, COBLL1, **ITGA4**, **PIEZO1**, POMGNT1*	Cardiac, Collagen processing, Cytoskeleton, Vascular
16	3	3	*COL28A1, PLS1, XIRP2*	Cytoskeleton, ECM structural
17	4	3	***COL11A2**, POMT1, TRPV6*	Collagen processing, Ion channel
18	7	5	***CAPN9**, FCGBP, GLRB, IRS1, SEC23A*	Collagen processing, Growth factor, Immune, Ion channel
19	6	6	***CDH26**, ITPR2, PDLIM5, SLC35D1, SOX9, XIRP2*	Ca2+ signaling, Cytoskeleton, Skeletal, TGF-β/BMP
20	3	3	***HMMR**, HSPG2, TTN*	ECM structural, Muscle
21	5	5	*CDC42BPB, **MMP7**, PCDHB10, **STAB2**, **TGM6***	Cytoskeleton, Immune
22	2	2	*MYOM2, SCN9A*	Ion channel, Muscle

^a^ Bold-formatted genes indicate genes belonging to the same pathways as the causative genes identified by WES in the three-generation Korean family.

**Table 6:** Ankle instability measurements in hEDS patients and the control group using manual ADT, stress ultrasonography, and stress radiography.

	hEDS Group (*n* = 22) ^a^	Control Group (*n* = 24) ^b^	Adjusted *p*-Value ^c^
Manual ADT (grade)	2.59 (2–3)	1.67 (1–3)	**<** **0** **.001**
Stress ultrasonography (mm)			
Resting ATFL length	19.55 (17.00–22.00)	18.45 (16.50–21.00)	**0** **.0** **13**
Resting ATFL height	0.95 (0.00–3.00)	0.21 (0.00–1.60)	**0** **.0** **20**
Stress ATFL length	21.18 (18.00–24.00)	19.63 (17.80–21.10)	**0** **.0** **10**
Stress ATFL height	0.18 (0.00–1.00)	0.10 (0.00–0.90)	0.304
Difference between resting and stress US			
ATFL length	1.64 (0.00–4.00)	1.18 (0.00–3.10)	**0** **.0** **34**
ATFL height	0.77 (0.00–3.00)	0.11 (0.00–0.70)	**0** **.0** **12**
ATFL length ratio (stress/resting)	1.08 (1.00–1.20)	1.07 (1.00–1.17)	**0** **.0** **43**
Stress Ankle X-ray			
Anterior talar translation (mm)	5.86	3.50	**<** **0** **.001**
Talar tilt (°)	7.68	4.29	**0** **.0** **12**

^a^ Values are presented as the mean and range. Boldface indicates statistically significant difference between groups (FDR-adjusted *p* < 0.05); ^b^ Control data were obtained from a previous cross-sectional cohort study conducted at the same institution using an identical assessment protocol [3] in 24 healthy young participants (age 20–40 years) without GJH (Beighton score < 5); ^c^ *p*-values were adjusted using the Benjamini–Hochberg false discovery rate (FDR) correction.

## Data Availability

The data presented in this study are available on request from the corresponding author. The data are not publicly available due to privacy and ethical restrictions related to the sensitive nature of patient genetic information and the conditions of the Institutional Review Board approval.

## Supplementary Materials

The following supporting information can be downloaded at: https://www.mdpi.com/article/10.3390/jcm15103881/s1, Supplementary File S1—Supplementary Note S1: Detailed description of criterion 2 for the diagnosis of hEDS: at least two of the following features must be present; Supplementary File S2—Supplementary Video S1: External rotation of the shoulder in a patient with hEDS; Supplementary File S3—Includes Supplementary Tables S1–S4 with captions; Table S1: Baseline characteristics of a three-generation Korean hEDS family; Table S2: Detailed information on three functionally impactful variants identified in the hEDS family set; Table S3: Detailed information on variants and genes derived from hEDS-related pathway analysis across 22 patients; Table S4: Detailed information on 114 variants associated with hEDS-related pathways across 22 patients.
