# Supplementary material for: Genetic Causes and Ankle Instability in Hypermobile Ehlers–Danlos Syndrome (hEDS): An Integrated Analysis Using Whole-Exome Sequencing and Stress Imaging
Source: J Clin Med. 2026 May 18;15(10):3881. doi: 10.3390/jcm15103881 (PMC13207121; doi:10.3390/jcm15103881)
Supplement: Supplementary file 1 [file jcm-15-03881-s001.zip › 4.Supplementary_file1_note.pdf]

Supplementary File 1. Detailed description of criterion 2 for the diagnosis of hEDS: at least two of the following features must be present.

Criterion 2: Two or more among the following features (A–C).

Feature A: systemic manifestations of a more generalized connective tissue disorder (a total of five must be present)

1. Unusually soft or velvety skin

2. Mild skin hyperextensibility

3. Unexplained striae such as striae distensae or rubrae at the back, groins, thighs, breasts and/or abdomen in adolescents, men or prepubertal women without a history of significant gain or loss of body fat or weight

4. Bilateral piezogenic papules of the heel

5. Recurrent or multiple abdominal hernia(s) (e.g., umbilical, inguinal, crural)

6. Atrophic scarring involving at least two sites and without the formation of truly papyraceous and/or hemosideric scars as seen in classical EDS

7. Pelvic floor, rectal, and/or uterine prolapse in children, men or nulliparous women without a history of morbid obesity or other known predisposing medical condition

8. Dental crowding and high or narrow palate

9. Arachnodactyly, as defined in one or more of the following: (i) positive wrist sign (Steinberg sign) on both sides; (ii) positive thumb sign (Walker sign)

on both sides

10. Arm span-to-height ≥ 1.05

11. Mitral valve prolapse (MVP) mild or greater based on strict echocardiographic criteria

12. Aortic root dilatation with Z-score > +2

Feature B: positive family history, with one or more first degree relatives

Feature C: musculoskeletal complications (must have at least one):

1. Musculoskeletal pain in two or more limbs, recurring daily for at least 3months

2. Chronic, widespread pain for ≥3 months

3. Recurrent joint dislocations or frank joint instability, in the absence of trauma (a or b)

    a. Three or more atraumatic dislocations in the same joint or two or more atraumatic dislocations in two different joints occurring at different times

    b. Medical confirmation of joint instability at two or more sites not related to trauma
